# Chromogranin A: a useful biomarker in castration-resistant prostate cancer

**DOI:** 10.1007/s00345-022-04248-0

**Published:** 2022-12-17

**Authors:** Guillaume Ploussard, François Rozet, Guilhem Roubaud, Trevor Stanbury, Paul Sargos, Morgan Roupret

**Affiliations:** 1 Department of Urology, Clinique La Croix du Sud, Quint-Fonsegrives, Institut Universitaire du Cancer de Toulouse (IUCT-O), Toulouse, France; 2 Institut Mutualiste Montsouris, Paris, France; 3 Department of Medical Oncology, Institut Bergonié, Bordeaux, France; 4 Pro-Pens, Antony, France; 5 Department of Radiotherapy, Institut Bergonié, Bordeaux, France; 6 GRC 5 Predictive Onco-Uro, AP-HP, Urology, Pitié-Salpêtrière Hospital, Sorbonne University, 75013 Paris, France

**Keywords:** Chromogranin A, Castration resistant prostate cancer, Neuroendocrine prostate cancer, Neuron-specific enolase

## Abstract

**Purpose:**

The natural history of prostate cancer (PC) almost always evolves to castration-resistant prostate cancer (CRPC) status, sometimes comprising pure or mixed neuroendocrine prostate cancers (NEPC) differentiation. In CRPC, monitoring using only prostate-specific antigen (PSA) is not optimal since neuroendocrine differentiated cells do not secrete PSA. Thus, monitoring with PSA and chromogranin A (CgA) may be useful. This review aims to evaluate evidence for the usefulness of CgA assessments during the monitoring of prostate cancer.

**Method:**

This review was based on three recent meta-analysis concerning CgA and prostate cancer. Further data were obtained from PubMed and Embase databases by searches using keywords, including chromogranin A and prostate cancer.

**Results:**

CgA levels remain largely unchanged during the early PC evolution. The development of NEPC is characterised by lower PSA secretion and increased CgA secretion. Data supporting the prognostic value of high CgA baseline levels for survival are contrasting and scarce. However, increasing CgA levels early during treatment of metastatic (m)CRPC suggests resistance to treatment and predicts shorter survival, particularly in men with high baseline levels of CgA levels. In men with mCRPC, the first-line chemotherapy may be more appropriate than other agents when baseline CgA levels are high. Also, increasing CgA levels during treatment may indicate disease progression and may warrant a change of therapy.

**Conclusion:**

CgA monitoring at baseline and regularly during mCRPC management may be useful for monitoring disease evolution. An increased CgA baseline levels and increasing CgA levels may assist physicians with choosing and modifying therapy.

## Introduction

Prostate cancers (PCs) are an androgen-driven disease and therefore require testosterone for growth [1]. However, almost all PCs progress to castration-resistant prostate cancer (CRPC). This resistance to castration is often driven by androgen receptor (AR) splice variants and AR point mutations or amplifications [2]. Neuroendocrine differentiation, although less frequent, provides an alternative AR-independent mechanism of evolution [3].

A large proportion of CRPCs are still driven by AR signalling [4]. Thus, AR targeted therapies remain appropriate in these tumours. In the metastatic setting, androgen deprivation therapy (ADT) combined with androgen-receptor (AR) targeted therapies, including abiraterone, apalutamide, and enzalutamide, is the standard first-line therapy [5]. However, neuroendocrine differentiation can emerge after AR inhibition that is less dependent on AR signalling. In men with mCRPC, 15–20% of tumours become AR independent [6]. This change manifests by a histological change, after exposure to multiple treatments, from prostate adenocarcinoma to neuroendocrine contingents.

The neuroendocrine prostate cancer (NEPC) phenotype includes both pure small cell carcinomas and mixed adenocarcinoma-neuroendocrine tumours [6]. Pure or dominant NEPC phenotypes, compared to the more common prostatic adenocarcinoma, often have visceral metastases, lytic bone lesions, relatively low serum prostate-specific antigen (PSA) levels, resists to castration, and could response to platinum-based chemotherapy [7]. NEPC diagnosis is essentially based on morphological characteristics and detection of neuroendocrine markers, including synaptophysin, chromogranin, and CD56 [4].

In patients with pure or mixed NEPC, monitoring using only PSA is not optimal since neuroendocrine differentiated cells do not secrete PSA. The impact of new generation imaging such as PSMA PET/CT could be useful for surveillance at the stage of NEPC. However, no specific study can provide guidance for routine PET/CT-based monitoring in that setting. Thus, prostatic tumours with neuroendocrine differentiated cells often remain undetected or are difficult to monitor by serum PSA analysis.

Chromogranin A (CgA) is the main component of secretory granules of neuroendocrine cells [8]. In neuroendocrine cells, CgA regulates the storage and secretion of hormones and neuropeptides and serves as a precursor to biologically active peptides [8–10]. Enzymatic cleavage of CgA generates biologically active peptides, including vasostatin, pancreastatin, WE14, catestatin, and serpinin. However, most CgA is secreted into the blood unchanged [8].

CgA blood levels may be elevated in patients with heart failure, renal failure, hypertension, sepsis and in those with various inflammatory disorders, including inflammatory bowel disorder and rheumatoid arthritis [10]. In patients with PCs, the increased levels of CgA suggest the presence and/or progression of neuroendocrine tumours or subpopulations. Neuroendocrine tumours have cells capable of producing, storing, and secreting CgA [9]. Two plausible mechanisms exist to explain the increased secretion of CgA in these patients, either increased neuroendocrine differentiation or an increased stress response in neuroendocrine cells, under the pressure of treatment.

Irrespective of the mechanism, CgA is a potential, and currently underused, biomarker in patients with NEPC or with neuroendocrine differentiation subpopulations [9, 11]. However, CgA as a biomarker, due to confounding non-neoplastic conditions, may be most useful relative to prior levels and not as an absolute value. CgA should be included as a tool for monitoring the evolution of PC: to identify the presence of neuroendocrine tumour subpopulations and to assist physicians with patient follow-up and treatment decisions. In this publication, we provide rational for using CgA as a biomarker in patients with CRPC for monitoring disease evolution and for guiding treatment.

## Methods

**Fig. 1:**
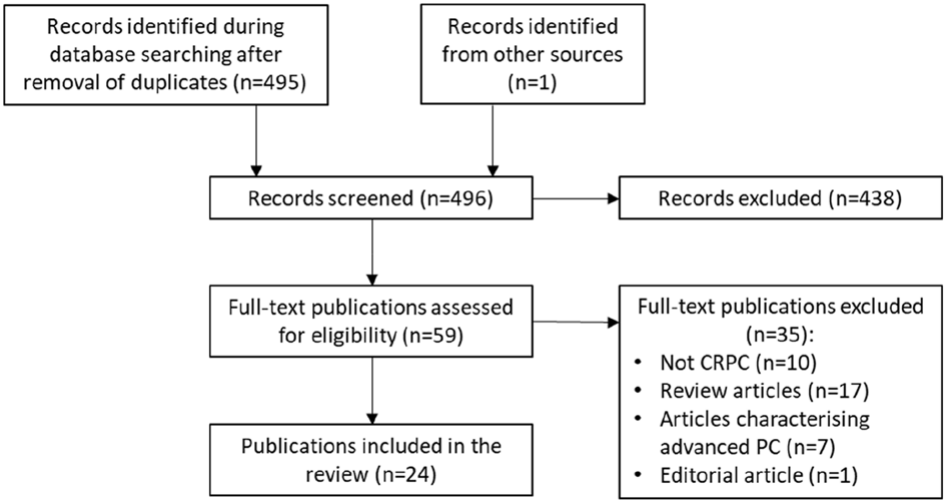
Flow diagram of the literature searches

To identify articles with relevant information, we performed a systematic literature research in the Pubmed and Embase electronic databases. The following search was performed in each database: prostate AND cancer AND chromogranin. Articles published prior to 2010 were not included. An article published by Aggarwal et al*.* was not identified during the PubMed and Embase database searches, but was considered relevant for the review [12]. The flow chart of the literature searches is shown in Fig. 1. Finally, 24 articles were included in this review (see Table 1).

**Table 1:** An overview of articles included in this review

Study	Men (*n*)	CgA/NSE (samples)	Prostate cancer stage	Relevant prior therapy	Therapy during or after sampling	Relevant analyses
Szarvas (2021) [13]	395	CgA and NSE (serum)	Localised and hormone-naïve PC and mCRPC	Localised PC: radical prostatectomy (*n* = 157); mCRPC: docetaxel (*n* = 95) or abiraterone with enzalutamide (*n* = 143)	Not applicable	Changes in CgA and NSE levels during treatment. Correlation of CgA and NSE levels with OS
Derlin (2020) [14]	50	CgA and NSE (serum)	mCRPC	Not relevant	^177^Lu-PSMA-617 RLT	Correlation between CgA and NSE levels with response to RLT and to PFS
Yordanova (2020) [15]	137	CgA (serum)	mCRPC	Not relevant	^177^Lu-PSMA-617 RLT	Correlation between CgA level with OS
Fan (2019) [16]	88	CgA and NSE (serum)	mCRPC	Sequences of abiraterone acetate and docetaxel-prednisone	Not applicable	Correlation of CgA and NSE levels with PFS and OS
Rathke (2019) [17]	100	CgA	mCRPC	Not relevant	^177^Lu-PSMA-617 RLT	Correlation between CgA levels and disease progression
Yang (2019) [18]	103	CgA and NSE (serum)	mCRPC	Not relevant	Prednisone with or without abiraterone	Change in CgA and NSE levels during treatment with abiraterone
Aggarwal (2018) [12]	202	CgA and NSE (tissue)	mCRPC	Antiandrogen treatment (*n* = 182); abiraterone and/or enzalutamide (*n* = 147)	Not applicable	Incidence of treatment-emergent small-cell NEPC and association with OS
Conteduca (2018) [2]	256	CgA (serum)	CRPC	Chemotherapy naïve	Abiraterone or enzalutamide	Association between baseline CgA levels and PFS and OS
Giridhar (2018) [19]	271	CgA (serum)	mCRPC	Not applicable	Not applicable	Association of CgA levels with OS
Thakur (2018) [20]	18	CgA and NSE (serum)	Chemotherapy-naïve mCRPC	Radiation (*n* = 10), prostatectomy (*n* = 7), enzalutamide (*n* = 3), and abiraterone (*n* = 5)	Docetaxel, prednisone, and pasireotide	Association between CgA and NSE levels and time to progression and OS
Dong (2017) [21]	115	CgA and NSE (serum)	Chemotherapy-naïve mCRPC	Abiraterone acetate (*n* = 48)	Abiraterone acetate (*n* = 67)	Assessing neuroendocrine differentiation during treatment
Niedworok (2017) [22]	237	CgA (serum and plasma)	Localized PC	Not applicable	Not applicable	Prognostic value of CgA levels for disease-specific survival
von Hardenberg (2017) [23]	52	CgA and NSE (serum)	mCRPC	Androgen derivation therapy (*n* = 52), docetaxel (*n* = 4), abiraterone acetate (*n* = 24), enzalutamide (*n* = 7)	Docetaxel	Dynamics of CgA and NSE levels during treatment. Correlation between levels with OS and PFS
Fan (2017) [24]	40	CgA and NSE (serum)	mCRPC	Chemotherapy (*n* = 18)	Abiraterone acetate	Prognostic value of changes in CgA and NSE levels after 3 months of treatment for PFS and OS
Heck (2017) [25]	45	CgA and NSE (serum)	mCRPC	Chemotherapy (*n* = 45)	Abiraterone	Prognostic value of CgA and NSE levels at baseline for PFS and OS during treatment
Angulo (2016) [26]	45	CgA (tissue)	Localised and advanced PC	Not applicable	Not applicable	Correlation between CgA levels and cancer-specific survival
Mahameddi (2016) [27]	30	CgA and NSE (serum)	Progressive CRPC	Prostatectomy/radiotherapy, and hormonal therapy	Docetaxel-prednisone and curcumin	Prognostic value of CgA and NSE baseline levels and OS
Von Hardenberg (2016) [28]	35	CgA and NSE (serum)	Chemotherapy-naïve CRPC	Abiraterone acetate (*n* = 16)	Docetaxel-based chemotherapy	Influence of abiraterone acetate treatment on CgA and NSE levels
Burgio (2014) [29]	48	CgA (serum)	mCRPC	Chemotherapy (*n* = 48)	Abiraterone acetate with prednisone	Predictive value of CgA baseline levels treatment response. Association between CgA baseline levels with PFS and OS
Mitsui (2015) [30]	16	CgA (tissue)	CRPC	Not applicable	Docetaxel, estramustine, and carboplatin	Change in CgA levels with disease evolution. Change in CgA levels with chemotherapy
Conteduca (2014) [31]	35	CgA (serum)	mCRPC	Docetaxel	Enzalutamide	Prognostic value of CgA levels at baseline for PFS and OS
Matei (2012) [32]	47	CgA (serum and tissue)	CRPC	Hormonal therapy with or without radiotherapy	Not applicable	To identify biomarkers of neuro-endocrine differentiation
Fléchon (2011) [33]	56	CgA and NSE (serum)	Progressive mCRPC	Hormonal therapy (*n* = 56); chemotherapy (*n* = 38)	Carboplatin and etoposide	CgA and NSE levels as predictors of treatment response and as prognostic markers for PFS and OS
Sarkar (2010) [34]	14	CgA (plasma)	mCRPC	Antiandrogenic treatment (*n* = 14)	Docetaxel	Prognostic value of CgA levels for OS. Association between CgA response and clinical response

The articles are described by the number of study participants, the type of CgA/NSE samples assessed, the prostate cancer stage, the relevant prior therapy, the therapy under study, and the relevant analyses for this review

*CgA* chromogranin A, *CRPC* castration-resistant prostate cancer, *mCRPC* metastatic castration-resistant prostate cancer, *NEPC* neuroendocrine prostate cancer, *NSE* neuron-specific enolase, *OS* overall survival, *PC* prostate cancer, *PFS* progression-free survival, *PSMA* prostate-specific membrane antigen, *RLT* radioligand therapy

## Results

### Biomarker levels during prostate cancer evolution

In general, CgA levels remain largely unchanged during the early evolution of PCs. As previously mentioned, neuroendocrine differentiated cells do not secrete PSA. Thus, CgA, as a biomarker, is expected to be more useful in patients with neuroendocrine differentiated cells that tends to emerge with castration resistance. This was confirmed in a recent study; CgA and neuron-specific enolase (NSE) levels, another neuroendocrine biomarker, were assessed in serum samples from 395 men at various stages of PC: 157 with hormone-naïve localised PC after radical prostatectomy and 238 with mCRPC (95 treated with first-line docetaxel and 143 treated with the first- or second-line abiraterone and/or enzalutamide) [13]. CgA and NSE levels were 2–3 times higher in patients with mCRPC than those with localised PC. Similarly, Niedworok et al*.* assessed whether CgA levels were associated with disease aggressiveness and oncologic outcomes such as long-term disease-specific survival [22]. CgA levels were significantly higher in patients with advanced disease compared with those with localised PC. Mitsui and his colleagues, in a retrospective analysis in 16 men, found that tissular CgA levels significantly increase from initial PC diagnosis to CRPC diagnosis [30].

NEPC is a highly aggressive disease characterised by lower PSA secretion and increased secretion of neuroendocrine biomarkers, including CgA, synaptophysin, and NSE. The emergence of NEPC during CRPC evolution is believed to be treatment related in up to 20% of patients [2, 35]. In the metastatic setting, most CRPC are still driven by the AR signalling pathway. However, PC phenotypes that are less reliant on AR signalling do develop after AR inhibition, including NEPC. The increased levels of CgA are believed to indicate the presence of neuroendocrine differentiated cells that secrete CgA.

### Prognostic value of baseline CgA levels on survival in patients with m0CRPC and mCRPC

Recently, two systematic reviews and meta-analyses explored the prognostic role of CgA as a biomarker in CRPC [36, 37]. Hong et al. specifically studied CgA in CRPC [36], while Liu et al*.* assessed CgA and NSE as biomarkers specifically in the more advanced mCRPC setting [37]. Both studies concluded that men with high baseline CgA levels tended towards shorter OS and PFS [36, 37]. At present, the data supporting the prognostic value of CgA levels at baseline for survival are contrasting and scarce. These findings are mainly based on small retrospective cohort and case-report studies.

The following studies, reported after 2010, found that high baseline CgA levels predicted shorter OS [2, 19, 20, 24, 25, 31, 32]. Three studies simultaneously assessed CgA and NSE levels as predictors of OS [13, 24, 25]. The combined analyses were performed to compensate for the heterogeneous secretion of the neuroendocrine biomarkers. Heck et al. assessed CgA and NSE baseline levels in 45 patients with mCRPC, after chemotherapy and before initiating abiraterone acetate [25]. CgA and NSE levels were assessed combined: either both low (CgA ≤ 85 ng/mL and NSE ≤ 16 ng/mL), both high, or one high and the one low. OS was significantly shorter when CgA and/or NSE baseline levels were high at baseline. The survival benefit was more pronounced when both CgA and NSE were low at baseline. Similarly, Fan et al. found that in 40 men with mCRPC, low levels of both CgA and NSE at baseline, before initiating abiraterone acetate, were associated with prolonged survival [24]. Interestingly, Szarvas et al*.* assessed baseline CgA and NSE levels in two mCRPC treatment cohorts: either before docetaxel (*n* = 95) or before abiraterone acetate/enzalutamide (*n* = 143) [13]. Higher baseline levels of CgA, but not NSE levels, were associated with shorter OS in both treatment cohorts: the association with CgA was more pronounced in the abiraterone acetate/enzalutamide cohort.

A further five studies focused on baseline CgA levels [2, 19, 20, 31, 32]. Giridhar et al. found that elevated CgA levels at baseline were significantly associated with shorter OS [19]. Conteduca et al. classified 35 mCRPC patients into 3 groups according to baseline CgA levels: < 120 ng/mL (*n* = 10), between 120 and 360 (*n* = 17), and ≥ 360 (*n* = 8). Baseline CgA level ≥ 360 ng/mL was a significantly predictor of shorter OS [31]. More recently, Conteduca et al*.* validated these results in a larger study (*n* = 256). Elevated baseline CgA levels were found to predict a shorter PFS and OS in patients with CRPC [2]. Matei et al. found that baseline CgA levels, using a 20 U/L cut-off, were not associated with OS [32]. However, elevated CgA levels, higher than ≥ 36 U/L, were significantly associated with shorter OS. Finally, the prognostic value of CgA was explored in a phase I trial assessing docetaxel-prednisone combined with pasireotide, a somatostatin receptor analogue, for treating men with chemotherapy-naïve mCRPC [20]. High baseline CgA levels, above 100 ng/mL, correlated with OS: hazard ratio of 1.07 (80% CI 1.02–1.12).

In contrast, the following studies found that baseline CgA levels were not prognostic for survival [26, 27, 29]. A study assessing docetaxel, prednisone, combined with curcumin for treating chemotherapy-naïve mCRPC found no association between elevated baseline levels of CgA, nor those of NSE, and OS [27]. Burgio et al*.* assessed the prognostic value of CgA levels in 48 patients with mCRPC treated with abiraterone acetate. CgA was not significantly associated with OS [29]. Similarly, Angulo and his co-workers did not observe a correlation with baseline CgA levels (tissular) and cancer-specific survival in 45 men with advanced PC [26].

It is important to note that we do not expect baseline CgA levels to be elevated in all men with mCRPC, only in those with pure or mixed NEPC. Indeed, a biopsy study of 202 patients with mCRPC, 148 that had progressed on prior treatment with abiraterone and/or enzalutamide, found that only 17% had small-cell NEPC subtype [12]. Detection of small-cell NEPC was significantly associated with shorter OS.

Overall, the data suggest that high baseline CgA levels, suggesting the presence of NEPC subtype, are associated with shorter OS in patients with mCRPC. However, the prognostic value of CgA may be limited by the extended survival due to recent therapeutic advances [38]. Thus, CgA, either alone or combined with NSE, may be useful to detect patients with neuroendocrine tumour subtypes that are associated with shorter survival and to predict response to certain treatments.

### Increasing CgA levels early during treatment predicts poor outcomes

There is an evidence to suggest that increasing CgA levels early during treatment may suggest resistance to treatment or neuroendocrine differentiation and consequently a shorter survival: particularly in patients with higher baseline CgA levels.

Increased baseline CgA levels above 360 ng/mL (3 time the upper limit of normal) predict early disease progression within the first 3 months of abiraterone treatment in patients with mCRPC [29]. Fan et al. assessed CgA and NSE levels in 40 men with mCRPC; increase in either CgA and/or NSE levels during the first 3 months of abiraterone was an independent predictor of poor survival: PSA PFS, radiographic PFS, and OS [24]. A recent study conducted by Szarvas et al. reported that in 143 men with mCRPC, treated with either abiraterone or enzalutamide, an increase in CgA levels of > 50% from baseline at 3 months was associated with shorter survival [13]. The association was even more pronounced in patients with high CgA levels at baseline. Interestingly, this association was not observed in the 95 patients being treated with docetaxel.

However, in contrast, von Hardenberg et al. reported that patients with an increase in CgA within the first and third cycle of docetaxel tended toward shorter OS (*p* = 0.055) and had significantly shorter PFS (*p* = 0.037) [23]. Similarly, a study reported by Sarkar et al*.* suggests that in CRPC men treated with docetaxel rising CgA levels predicts a poor prognosis, while a lowering CgA levels is probably associated with clinical response to treatment [34].

In men with mCRPC treated with abiraterone or enzalutamide, increasing CgA levels, particularly in those with high CgA levels at baseline, suggests resistance to treatment and probably the presence of neuroendocrine differentiation. During docetaxel, lowering CgA levels may indicate a response to treatment with increasing levels suggesting neuroendocrine differentiation with a poor survival prognosis.

### CgA as a biomarker of response to treatment

#### Response to chemotherapy

Several studies have evaluated changes in CgA levels, and NSE levels, during chemotherapy [27, 33, 39]. A study assessed carboplatin combined with etoposide in men with mCRPC and with increased CgA and/or NSE levels at baseline, indicating the presence of visceral metastasis or neuroendocrine differentiation [33]. At baseline, 64% of them had CgA levels, 36% had NSE levels, and 21% had both CgA and NSE levels ≥ 1.5 times upper limit of normal. During carboplatin combined with etoposide, CgA levels decreased by > 50% in 7%, were stable in 31%, and increased by > 25% in 62% of men. Similarly, NSE levels decreased by > 50% in 31%, were stable in 25%, and increased by > 25% in 44% of patients. Similarly, Culine et al. assessed cisplatin and docetaxel in 41 men with mCRPC with elevated CgA and/or NSE baseline levels: after at most six 3-week cycles, 33% had a neuroendocrine response (NSE and/or CgA decreased by > 50%) [39]. Similarly, in a study assessing docetaxel, prednisone, combined with curcumin for treating chemotherapy-naïve mCRPC, CgA levels decreased by > 50% in 7%, was stable in 40%, and increased by > 25% in 53% of men [27]. NSE levels decreased by > 50% in 30%, was stable in 60%, and increased by > 25% in 10% of men. Finally, Mitsui and his colleagues found that in men with CRPC, tissular CgA expression significantly decreased after 2 cycles of docetaxel, estramustine, and carboplatin [30]. Thus, there is evidence to suggest that chemotherapy lowers CgA and NSE levels in selected mCRPC patients.

#### Response to new generation hormonal therapies

Studies have shown that abiraterone does not drive neuroendocrine differentiation and is not directly associated with increasing of CgA levels [18, 21, 28]. CgA levels were assessed during abiraterone treatment in 34 men with chemotherapy naïve mCRPC. After 6 months of abiraterone, 17 men (50%) had increased CgA levels and 17 (50%) had decreased CgA levels [21]. At abiraterone treatment failure, 14 men (41%) had increased CgA levels and 20 (59%) had decreased CgA levels. There was no significant difference in between CgA levels at baseline and at abiraterone treatment failure. A study reported by Yang et al*.* retrospectively compared CgA and NSE levels in 103 men with mCRPC: 71 treated with abiraterone with prednisone and 32 with prednisone alone (control group). The CgA and NSE levels in the groups were not significantly different prior to treatment [18]. However, after 6 months, significantly more men in the control group had elevated CgA and NSE levels. These data show that treatment with abiraterone does not induce increases in CgA and NSE levels.

A study reported by von Hardenberg et al. assessed CgA levels in 35 men with chemotherapy naïve CRPC [28]. Among these, 16 men had previously treated with abiraterone. Overall, in the 35 men baseline levels of CgA were abnormal in 20 men. In multivariate analysis, treatment with abiraterone and duration of treatment were not associated with the abnormal levels of CgA. Interestingly, Szarvas et al. reported that in mCRPC patients treated with the first- or second-line abiraterone and/or enzalutamide a > 50% increase in CgA from baseline levels at 3 months was associated with shorter survival, particularly when baseline CgA levels were already high [13]. These results suggest that increased CgA levels, at 3 months, are probably due to neuroendocrine differentiation and suggest the inefficacy of abiraterone in this population.

#### Response to prostate-specific membrane antigen (PSMA)-targeted radioligand therapy (RLT)

Prostate-specific membrane antigen (PSMA)-targeted radio-ligand therapy (RTL) is one of the last treatment options for men with mCRPC. Three recent studies assessed whether CgA levels could predict response to PSMA-targeted RTL: ^177^Lu-PSMA-617 RLT [14, 15, 17]. Derlin et al*.* found that neither CgA nor NSE levels predicted treatment failure nor early disease progression in 50 men undergoing PSMA-targeted RTL [14]. Similarly, Yordanova et al*.* found no significant correlation between baseline CgA levels and OS in 137 men treated with PSMA-target RTL [15]. Although not significant, men with normal baseline CgA (limit of 100 ng/mL) survived for 72 weeks compared to 60 weeks in those with elevated CgA levels. Furthermore, in the men with elevated CgA levels at baseline (*n* = 69), those with stable CgA levels after treatment survived for 93 weeks, those with decreased CgA levels for 61 weeks, and those with increased CgA levels for 30.3 weeks. Finally, Rathke et al*.* reported that progressive disease was significantly associated with elevated CgA levels prior to RTL [17]. Interestingly, men with elevated CgA levels at baseline had a significantly increased risk of liver metastasis.

### CgA levels as a guide to treatment sequencing in mCRPC

**Fig. 2:**
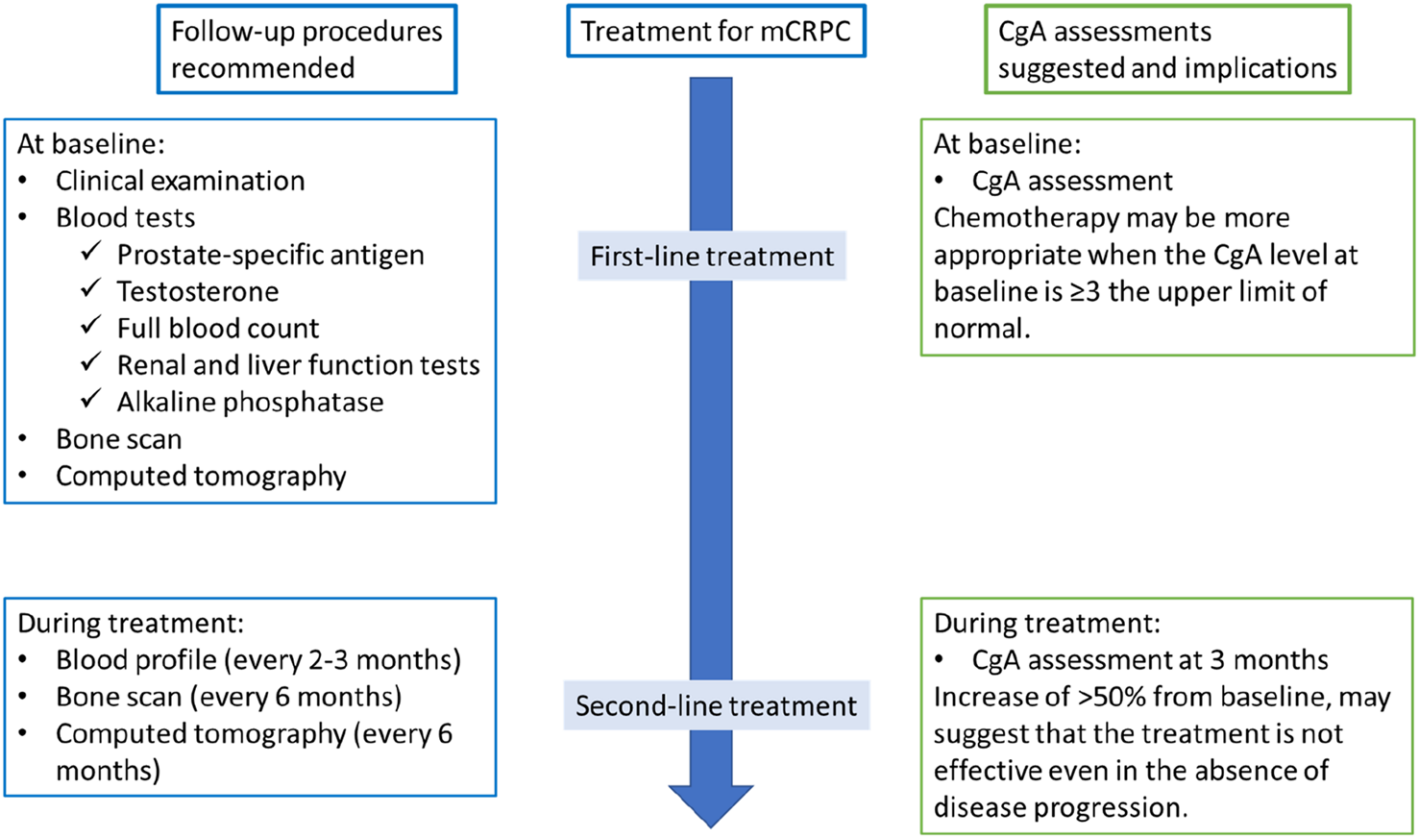
Suggested CgA monitoring during the therapeutic management of men with mCRPC

Due to heterogeneity of mCRPC, with a variety of AR-targeted and non-AR-target treatment options, CgA levels may be useful for guiding the sequencing of treatments. A recent study assessed whether CgA and NSE levels could be used to guide treatment sequencing of abiraterone acetate and combined docetaxel-prednisone in mCRPC [16]. Men with elevated levels of either CgA or NSE survived significantly longer when docetaxel-prednisone was administered before abiraterone acetate. Men without elevated biomarkers had similar survival outcomes with the sequences. Thus, chemotherapy may be a better option in mCRPC patients with high CgA levels and NSE levels at baseline or with increasing levels during the first 3 months of abiraterone treatment [24]. Our suggested CgA assessments and implications for treatment are shown in Fig. 2.

One important limitation to highlight is that the management landscape of advanced prostate cancer patients has completely changed during the last decade. Thus, the selected regimen in included studies could not reflect the current standard of care and could impact on proposed follow-up flow chart.

## Conclusion

CgA appears as a valuable biomarker in PC, particularly in CRPC, to detect neuroendocrine differentiated tumours or subpopulations. Furthermore, CgA may be useful to guide treatment sequencing in men with mCRPC. Thus, CgA may be useful prior to the first-line treatment of men with mCRPC and then regularly to identify early treatment resistance and initiate therapeutic line changes. There is a current paucity of data concerning the use of CgA. The systematic inclusion of CgA assessments in clinical studies would provide valuable and much needed data concerning the role of CgA as a biomarker in PC.

## References

[1] Pienta KJ, Bradley D (2006). Mechanisms underlying the development of androgen-independent prostate cancer. Clin Cancer Res.

[2] Conteduca V, Scarpi E, Salvi S, Casadio V, Lolli C, Gurioli G (2018). Plasma androgen receptor and serum chromogranin A in advanced prostate cancer. Sci Rep.

[3] Aggarwal R, Romero GR, Friedl V, Weinstein A, Foye A, Huang J (2021). Clinical and genomic characterization of Low PSA Secretors: a unique subset of metastatic castration resistant prostate cancer. Prost Cancer Prostatic Dis.

[4] Vlachostergios PJ, Puca L, Beltran H (2017). Emerging variants of castration-resistant prostate cancer. Curr Oncol Rep.

[5] Rozet F, Mongiat-Artus P, Hennequin C, Beauval JB, Beuzeboc P, Cormier L, Fromont-Hankard G, Mathieu R, Ploussard G, Renard-Penna R, Brenot-Rossi I, Bruyere F, Cochet A, Crehange G, Cussenot O, Lebret T, Rebillard X, Soulié M, Brureau L, Méjean A (2020). Recommandations françaises du Comité de cancérologie de l’AFU—actualisation 2020–2022: cancer de la prostate. Assoc Française d'Urologie (AFU).

[6] Yamada Y, Beltran H (2021). Clinical and biological features of neuroendocrine prostate cancer. Curr Oncol Rep.

[7] Vlachostergios PJ, Papandreou CN (2015). Targeting neuroendocrine prostate cancer: molecular and clinical perspectives. Front Oncol.

[8] Laguerre F, Anouar Y, Montero-Hadjadje M (2020). Chromogranin A in the early steps of the neurosecretory pathway. IUBMB Life.

[9] Corti A, Marcucci F, Bachetti T (2018). Circulating chromogranin A and its fragments as diagnostic and prognostic disease markers. Pflugers Arch.

[10] Mahata SK, Corti A (2019). Chromogranin A and its fragments in cardiovascular, immunometabolic, and cancer regulation. Ann N Y Acad Sci.

[11] Gut P, Czarnywojtek A, Fischbach J, Baczyk M, Ziemnicka K, Wrotkowska E (2016). Chromogranin A—unspecific neuroendocrine marker. Clinical utility and potential diagnostic pitfalls. Arch Med Sci.

[12] Aggarwal R, Huang J, Alumkal JJ, Zhang L, Feng FY, Thomas GV (2018). Clinical and genomic characterization of treatment-emergent small-cell neuroendocrine prostate cancer: a multi-institutional prospective study. J Clin Oncol.

[13] Szarvas T, Csizmarik A, Fazekas T, Hüttl A, Nyirády P, Hadaschik B (2021). Comprehensive analysis of serum chromogranin A and neuron-specific enolase levels in localized and castration-resistant prostate cancer. BJU Int.

[14] Derlin T, Werner RA, Lafos M, Henkenberens C, von Klot CAJ, Sommerlath Sohns JM (2020). Neuroendocrine differentiation and response to PSMA-targeted radioligand therapy in advanced metastatic castration-resistant prostate cancer: a single-center retrospective study. J Nucl Med.

[15] Yordanova A, Linden P, Hauser S, Feldmann G, Brossart P, Fimmers R (2020). The value of tumor markers in men with metastatic prostate cancer undergoing [(177) Lu]Lu-PSMA therapy. Prostate.

[16] Fan L, Yang Y, Chi C, Ma X, Wang R, Gong Y (2019). Neuroendocrine differentiation markers guide treatment sequence selection in metastatic castration-resistant prostate cancer. Prostate.

[17] Rathke H, Holland-Letz T, Mier W, Flechsig P, Mavriopoulou E, Röhrich M (2020). Response prediction of (177)Lu-PSMA-617 radioligand therapy using prostate-specific antigen, chromogranin A, and lactate dehydrogenase. J Nucl Med.

[18] Yang K, Li T, Gao Z, Zhang W (2019). Effect of abiraterone combined with prednisone on serum CgA and NSE in metastatic castration-resistant prostate cancer without previous chemotherapy. Trop J Pharm Res.

[19] Giridhar KV, Sanhueza C, Hillman DW, Alkhateeb H, Carlson R, Tan W (2018). Serum chromogranin-A-based prognosis in metastatic castration-resistant prostate cancer. Prostate Cancer Prostatic Dis.

[20] Thakur MK, Heilbrun L, Dobson K, Boerner J, Stark K, Li J (2018). Phase I trial of the combination of docetaxel, prednisone, and pasireotide in metastatic castrate-resistant prostate cancer. Clin Genitourin Cancer.

[21] Dong B, Fan L, Wang Y, Chi C, Ma X, Wang R (2017). Influence of abiraterone acetate on neuroendocrine differentiation in chemotherapy-naive metastatic castration-resistant prostate cancer. Prostate.

[22] Niedworok C, Tschirdewahn S, Reis H, Lehmann N, Szücs M, Nyirády P (2017). Serum chromogranin A as a complementary marker for the prediction of prostate cancer-specific survival. Pathol Oncol Res.

[23] von Hardenberg J, Schwartz M, Werner T, Fuxius S, Muller M, Frangenheim T (2017). Prospective evaluation of neuromediator dynamics in castration-resistant prostate cancer patients during docetaxel. Anticancer Res.

[24] Fan L, Wang Y, Chi C, Pan J, Xun S, Xin Z (2017). Chromogranin A and neurone-specific enolase variations during the first 3 months of abiraterone therapy predict outcomes in patients with metastatic castration-resistant prostate cancer. BJU Int.

[25] Heck MM, Thaler MA, Schmid SC, Seitz AK, Tauber R, Kübler H (2017). Chromogranin A and neurone-specific enolase serum levels as predictors of treatment outcome in patients with metastatic castration-resistant prostate cancer undergoing abiraterone therapy. BJU Int.

[26] Angulo JC, Redondo C, Sánchez-Chapado M, Colás B, Ropero S, López JI (2016). Survival predictors in patients with prostate adenocarcinoma with hormonal blockade. Pathol Res Pract.

[27] Mahammedi H, Planchat E, Pouget M, Durando X, Curé H, Guy L (2016). The new combination docetaxel, prednisone and curcumin in patients with castration-resistant prostate cancer: a pilot phase II study. Oncology.

[28] von Hardenberg J, Schwartz M, Werner T, Fuxius S, Müller M, Bolenz C (2016). Influence of abiraterone acetate on circulating neuromediators in chemotherapy-naïve castration-resistant prostate cancer. Prostate.

[29] Burgio SL, Conteduca V, Menna C, Carretta E, Rossi L, Bianchi E (2014). Chromogranin A predicts outcome in prostate cancer patients treated with abiraterone. Endocr Relat Cancer.

[30] Mitsui Y, Arichi N, Hiraki M, Harada Y, Yasumoto H, Shiina H (2015). Tissue chromogranin A expression during prostate cancer progression: prediction of chemosensitivity. Urol J.

[31] Conteduca V, Burgio SL, Menna C, Carretta E, Rossi L, Bianchi E (2014). Chromogranin A is a potential prognostic marker in prostate cancer patients treated with enzalutamide. Prostate.

[32] Matei DV, Renne G, Pimentel M, Sandri MT, Zorzino L, Botteri E (2012). Neuroendocrine differentiation in castration-resistant prostate cancer: a systematic diagnostic attempt. Clin Genitourin Cancer.

[33] Fléchon A, Pouessel D, Ferlay C, Perol D, Beuzeboc P, Gravis G (2011). Phase II study of carboplatin and etoposide in patients with anaplastic progressive metastatic castration-resistant prostate cancer (mCRPC) with or without neuroendocrine differentiation: results of the French Genito-Urinary Tumor Group (GETUG) P01 trial. Ann Oncol.

[34] Sarkar D, Singh SK, Mandal AK, Agarwal MM, Mete UK, Kumar S (2010). Plasma chromogranin A: clinical implications in patients with castrate resistant prostate cancer receiving docetaxel chemotherapy. Cancer Biomark.

[35] Zhang C, Qian J, Wu Y, Zhu Z, Yu W, Gong Y (2021). Identification of novel diagnosis biomarkers for therapy-related neuroendocrine prostate cancer. Pathol Oncol Res.

[36] Hong P, Guo RQ, Song G, Yang KW, Zhang L, Li XS (2018). Prognostic role of chromogranin A in castration-resistant prostate cancer: a meta-analysis. Asian J Androl.

[37] Liu Y, Zhao S, Wang J, Zhu Z, Luo L, Li E (2019). Serum neuroendocrine markers predict therapy outcome of patients with metastatic castration-resistant prostate cancer: a meta-analysis. Urol Int.

[38] Appetecchia M, Lauretta R, Sperduti I, Gallucci M (2018). Chromogranin A as a biomarker for prostate cancer: is it actually relevant for clinical practice?. Future Oncol.

[39] Culine S, El Demery M, Lamy PJ, Iborra F, Avances C, Pinguet F (2007). Docetaxel and cisplatin in patients with metastatic androgen independent prostate cancer and circulating neuroendocrine markers. J Urol.

